# A versatile model with three-dimensional triangular lattice for unconventional transport and various topological effects

**DOI:** 10.1093/nsr/nwad114

**Published:** 2023-04-25

**Authors:** Jing-Yang You, Gang Su, Yuan Ping Feng

**Affiliations:** Department of Physics, National University of Singapore, Singapore 117551, Singapore; Kavli Institute for Theoretical Sciences, and CAS Center for Excellence in Topological Quantum Computation, University of Chinese Academy of Sciences, Beijing 100190, China; Department of Physics, National University of Singapore, Singapore 117551, Singapore; Centre for Advanced 2D Materials, National University of Singapore, Singapore 117546, Singapore

**Keywords:** three-dimensional triangular lattice, different topological phases, various topological effects, Berry curvature

## Abstract

The finite Berry curvature in topological materials can induce many subtle phenomena, such as the anomalous Hall effect (AHE), spin Hall effect (SHE), anomalous Nernst effect (ANE), non-linear Hall effect (NLHE) and bulk photovoltaic effects. To explore these novel physics as well as their connection and coupling, a precise and effective model should be developed. Here, we propose such a versatile model—a 3D triangular lattice with alternating hopping parameters, which can yield various topological phases, including kagome bands, triply degenerate fermions, double Weyl semimetals and so on. We reveal that this special lattice can present unconventional transport due to its unique topological surface states and the aforementioned topological phenomena, such as AHE, ANE, NLHE and the topological photocurrent effect. In addition, we also provide a number of material candidates that have been synthesized experimentally with this lattice, and discuss two materials, including a non-magnetic triangular system for SHE, NLHE and the shift current, and a ferromagnetic triangular lattice for AHE and ANE. Our work provides an excellent platform, including both the model and materials, for the study of Berry-curvature-related physics.

## Teaser

This work presents a versatile model with 3D triangular lattice, which can produce a variety of intriguing topological band structures and various topological phenomena and is applicable to many real materials.

## INTRODUCTION

In the past decade, the study of the topological electronic structure of crystalline materials has emerged as a particularly important research field in condensed matter physics and materials science [1,2]. The quantum Hall effect (QHE) [3] is the first instance of a topologically non-trivial state and hence opens the field of topological state research. Charged particles undergo deflected motion under a strong external magnetic field, creating a voltage that drives electrons to fill in discrete Landau levels, giving rise to the quantized Hall conductance σ_*xy*_ = *Ce*^2^/*h* with *C* being an integer (Chern number [4,5]). Nonetheless, QHE can be achieved without the external magnetic field, i.e. quantum anomalous Hall effect (QAHE) [6]. AHE has an intrinsic origin because of the spin-dependent band structure, which can be described in terms of the Berry phase in momentum space [7]. This effect comes from spin-orbit coupling (SOC), resulting in opposite motions of spin-up and spin-down electrons. The populations of spin-up and spin-down electrons in a ferromagnetic metal will become unbalanced as a result of spontaneous magnetization, which will ultimately generate AHE. In a time-reversal (TR) symmetry invariant system, the numbers of electrons traveling in opposing directions with different spins is equal, and as a result, there is non-zero spin Hall conductance without charge Hall conductance, called the quantum spin Hall (QSH) state [8,9]. The edge states of topological insulators including QAH and QSH effects are resistant to impurities or disorders because electron backscattering is prevented by TR symmetry, giving rise to dissipationless transport [10,11].

The amazing finding of topological insulators provided the initial impetus, but soon attention was turned to topological semimetals and metals, where two or more bands cross at specific momenta, as was the case with the identification of Weyl [12–14] and Dirac [15–17] semimetals. There are surface states that connect the projections of Weyl points with opposite charges in the form of Fermi arcs. Owing to the lack of partial scattering channels, unconventional transport can occur through an open Fermi arc [18]. In addition, non-centrosymmetric crystals can display the bulk photovoltaic effect (BPVE) under homogeneous illumination [19]. When light is absorbed, this non-linear optical response involves the production of a photocurrent or photovoltage. BPVE contains a ‘circular’ part that shifts sign in accordance with the helicity of light and a ‘linear’ component that only manifests itself with linearly polarized or unpolarized light. The former is best known for the quantized circular photogalvanic effect (CPGE) [20,21], which directly gauges topological charge of Weyl points, and the intrinsic contribution to the latter is the shift current owing to interband absorption [22–24].

The aforementioned topological states have a common microscopic origin, namely Berry curvature Ω(**k**) [25], which is a band structure characteristic of solid materials. Since the Berry curvature in a non-magnet with TR symmetry is odd in momentum space, its integral weighted by the equilibrium Fermi distribution is compelled to disappear, resulting in the disappearance of the anomalous Hall conductivity (AHC). Although a TR invariant system lacks transverse conductivity, it can nevertheless have transverse spin current (spin Hall conductivity (SHC)), which is attributed to the spin Berry curvature [10,26,27]. In addition, the Hall-like current can also exist in second-order response to the external electric field in a class of inversion symmetry breaking and TR invariant materials, producing a nonlinear Hall effect (NLHE) originating from the Berry curvature dipole (BCD) in momentum space [28]. Along with AHE, the anomalous Nernst effect (ANE) [29,30] caused by a temperature gradient in a TR-breaking system like a ferromagnet is also of great interest. ANE is dominated by the Berry curvature at the Fermi level in contrast to AHE that is dictated by the sum of Berry curvatures of all occupied states, thus supplying distinct detection of Berry curvatures near *E_F_* and topological properties. Anomalous Nernst conductivity (ANC) is proportional to the differential of AHC, and they are connected at low temperatures by the Mott relations [31,32].

Given these exotic topological states and corresponding responses related to the Berry curvature, a natural question is whether we can identify a real and straightforward lattice with a non-centrosymmetric crystal that can act as a good platform for exploring these phenomena and their correlation or coupling. Here, we suggest a versatile model with a three-dimensional (3D) triangular lattice with alternating hopping parameters, which is a chiral structure without inversion and mirror symmetries. We discover that this simple lattice model can produce many fascinating topological phases, such as kagome bands, triply degenerate fermions, double Weyl semimetals and so on. The Weyl points with opposite chiralities are well separated in energy as a result of the inversion asymmetric structure, leading to a distinctive distribution of topological Fermi arc surface states, which provides excellent prospects for surface unconventional transport. In addition, this lattice can further generate NLHE and shift current BPVE. We conclude by providing two examples of experimentally synthesized materials that display a 3D triangular lattice and band structures with various topological phases. A non-magnetic 3D triangular lattice is investigated for topological surface states, SHE, NLHE and the shift current, and a ferromagnetic one is discussed for AHE and ANE. This work not only provides a simple and effective lattice model to explore various topological states and their corresponding responses to electric field and light, but also covers numerous genuine material candidates for further investigation and device application.

## MODEL WITH A 3D TRIANGULAR LATTICE AND DIFFERENT TOPOLOGICAL PHASES

**Figure 1.**
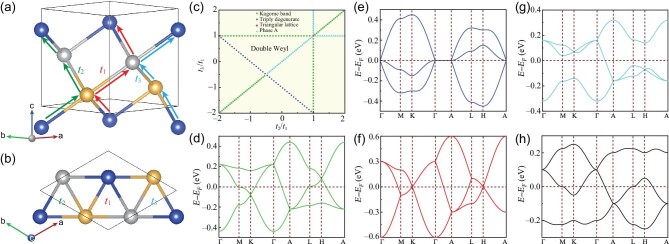
(a) Side and (b) top views of the 3D triangular lattice with distinct nearest-neighboring hoppings *t*_1_, *t*_2_ and *t*_3_, indicated by red, green and blue, respectively, where the yellow, silver and blue balls are at the heights of *c*/6, *c*/2 and 5*c*/6, respectively. (c) Phase diagram of the 3D triangular lattice in the *t*_2_/*t*_1_ and *t*_3_/*t*_1_ parameter spaces. Band structures corresponding to the different topological phases in (c): (d) kagome bands with *t*_2_/*t*_1_ = 1 and *t*_3_/*t*_1_ = 0.2, (e) triply degenerate fermions with *t*_2_/*t*_1_ = −1.5 and *t*_3_/*t*_1_ = 0.5, (f) folded triangular lattice bands with *t*_2_/*t*_1_ = *t*_3_/*t*_1_ = 1, (g) phase A bands with *t*_2_/*t*_1_ = *t*_3_/*t*_1_ = 0.3 and (h) double Weyl fermions with *t*_2_/*t*_1_ = −*t*_3_/*t*_1_ = −0.5. In all cases, *t*_1_ is taken as 0.1.

A 3D triangular lattice can be obtained by translating two atoms in a $\sqrt{3}\times \sqrt{3}$ supercell of a 2D triangular lattice containing three atoms along the *z* axis (out-of-plane direction) by *c*/3 and 2*c*/3, respectively, as shown in Fig. 1(a) and (b). Here we distinguish three different nearest-neighboring (NN) hoppings by *t*_1_, *t*_2_ and *t*_3_. The hopping parameters may be adjusted by light of different wavelengths in the ultracold atoms [33]. Here, we focus on the electronic systems. The hopping parameters of the 3D triangular lattice can be adjusted similarly to those of 2D lattices, since the hoppings inside each triangle are the same while those of different triangles differ. As a result, alternative hopping parameters can be physically realized by a 2D periodic potential. In a real material system, three equilateral triangles projected onto the *x*–*y* plane have uneven side lengths, resulting in the different hopping parameters, which can be further enhanced by biaxial strain or pressure. On the other hand, the triangular cavity of the 3D triangular lattice in a real system contains a variety of additional atoms that act as a periodic potential and alter the hopping parameters. We may also change the hopping parameters in real-world materials by doping the triangular cavity with various atoms at different concentrations. Therefore, the 3D triangular lattice can only be found in space groups (SGs) No. 154, No. 152, No. 145 and No. 144, which are chiral crystals without inversion and mirror symmetries.

We consider the Hamiltonian with NN hoppings on the 3D triangular lattice


(1) \begin{eqnarray*}
H &=&-\sum _{\langle ij \rangle \alpha s}t_sc^{\dagger }_{i\alpha }c_{j\alpha }+i\lambda \sum _{\langle ij \rangle \alpha \beta }(\mathbf {E}_{ij}\times \mathbf {R}_{ij}) \\
&& \cdot\, \mathbf {\sigma }_{\alpha \beta }c^{\dagger }_{i\alpha }c_{j\beta } +\,\mathbf {m}\cdot \mathbf {\sigma }\otimes \mathbf {I}_{3\times 3},
\end{eqnarray*}

where *t_s_* represents different NN hoppings as shown in Fig. 1(a) and the second and third terms are TR-invariant spin-orbit interactions and the Zeeman term that breaks TR symmetry, with **E**_*ij*_ the electric field from neighboring ions experienced along **R**_*ij*_, **m** the magnetic field and **I**_3 × 3_ the unit matrix of rank 3. First, let us consider a non-magnetic case with negligible SOC strength (λ = *m* = 0); in momentum space, Equation (1) becomes


(2) \begin{eqnarray*}
&& H_{\mathbf {k}}\!=\!-\! \sum _{u=1,2,3}t_u[{\rm cos}(\mathbf {k}\cdot \ell _u)M_u-{\rm sin}(\mathbf {k}\cdot \ell _u)N_u] \\
&&\quad -\sum _{u\ne v\ne w}t_u[{\rm cos}(\mathbf {k}\cdot \ell _v)M_w-{\rm sin}(\mathbf {k}\cdot \ell _v)N_w],\\
\end{eqnarray*}

where ℓ_1_ = (**a**_1_ − **a**_2_ + **a**_3_)/3 with **a_1_** = *a*(1, 0, 0), $\mathbf {a_2}=a(-1/2, \sqrt{3}/2, 0)$ and **a_3_** = *c*(0, 0, 1), ℓ_2_ = ( − 2**a**_1_ − **a**_2_ + **a**_3_)/3 and ℓ_3_ = (**a**_1_ + 2**a**_2_ + **a**_3_)/3 are the displacements from one site to its upper three NN sites, and


\begin{eqnarray*}
M_1 &=&\Bigg({\begin{array}{ll}\sigma _x &\quad 0\\
0 & \quad 0 \end{array}\Bigg)},\quad N_1= \Bigg( {\begin{array}{ll} \sigma _y &\quad 0\\
0 &\quad 0 \end{array} \Bigg)}, \\
M_2 &=& \Bigg({\begin{array}{ll} 0 & \quad 0\\
0 & \quad \sigma _x \end{array} \Bigg)}, \quad N_2= \Bigg({\begin{array}{lc} 0 & \quad 0\\
0 &\quad \sigma _y \end{array} \Bigg)}, \\
M_3 &=& \Bigg({\begin{array}{lcc} 0 &\quad 0 &\quad 1\\
0 &\quad 0 &\quad 0 \\
1 & \quad 0 &\quad 0 \end{array} \Bigg) },\quad N_3 = \Bigg({\begin{array}{lcc}
0 &\quad 0 &\quad i\\
0 &\quad 0 &\quad 0\\
-i &\quad 0 &\quad 0 \end{array} \Bigg)},
\end{eqnarray*}

with σ_*x*/*y*/*z*_ Pauli matrices.

In Fig. 1(c), we illustrate the phase diagram (assuming that *t*_1_ ≠ 0) for a 3D triangular lattice in the *t*_1_, *t*_2_ and *t*_3_ parameter spaces. It should be observed that, for *t*_3_/*t*_1_ = *t*_2_/*t*_1_ < −0.5 or *t*_2_/*t*_1_(*t*_3_/*t*_1_) = 1 and *t*_3_/*t*_1_(*t*_2_/*t*_1_) < 1 or *t*_3_/*t*_1_ = *t*_2_/*t*_1_ > 1 at *k_z_*(*k*_3_) = 0 and π, 2D kagome bands are reproduced, including the ‘flat’ band, Weyl points and Van Hove singularities, as shown in Fig. 1(d). Only when *t*_2_ or *t*_3_ is zero will the ‘flat’ band be completely flat without dispersion. In the case of *t*_2_/*t*_1_ + *t*_3_/*t*_1_ = 1, triply degenerate fermions appear at Γ and A points, as given in Fig. 1(e). For a completely ideal case with *t*_1_ = *t*_2_ = *t*_3_, the three bands are simply folded from a single band of perfect triangular lattice (see Fig. 1(f)). As for −0.5 < *t*_3_/*t*_1_ = *t*_2_/*t*_1_ < 1 or *t*_2_/*t*_1_(*t*_3_/*t*_1_) = 1 and *t*_3_/*t*_1_(*t*_2_/*t*_1_) > 1, a topological phase (here designated as phase A) arises, as seen in Fig. 1(g), which exhibits some similarities to, but also differs from, the kagome bands in Fig. 1(d). In kagome bands, the single Weyl points carrying a charge |*C*| = 1 at *K*/*K*′ (*H*/*H*′) are paired with the double Weyl with |*C*| = 2 at the A (Γ) point, and they have opposite chiralities, while for phase A in Fig. 1(g), the single Weyl points at *K*/*K*′ (*H*/*H*′) are paired with the double Weyl at the Γ (A) point. Different pairing positions of opposite chiral fermions will lead to diverse Fermi arcs projected onto the surface, so we classify them into two categories here. In addition to some special parameters mentioned above, for more arbitrary *t*_2_ and *t*_3_ (blank area in Fig. 1(c)), the system enters the double Weyl semimetallic phase, as shown in Fig. 1(h). These phases share a common feature in that the band structure in the *k_z_* = π plane is reversed compared to that in the *k_z_* = 0 plane because of a π-phase difference. The three-fold screw symmetry in a 3D triangular lattice is directly responsible for stabilizing these Weyl points at high-symmetry momenta (see the detailed symmetry analysis in the online supplementary material). The topological charges of these Weyl points, as sinks or sources of Berry flux, can be determined either by integrating Berry curvatures or by analyzing the rotational eigenvalues [34]. The single Weyl point at the K or H point has linear dispersion, so its topological charge is 1; while the double Weyl point at the Γ point is quadratically dispersive, so its topological charge is 2. The topological charges 2 of a double Weyl point are protected by the joint of the screw and time-reversal symmetries.

### Non-local transport

**Figure 2.**
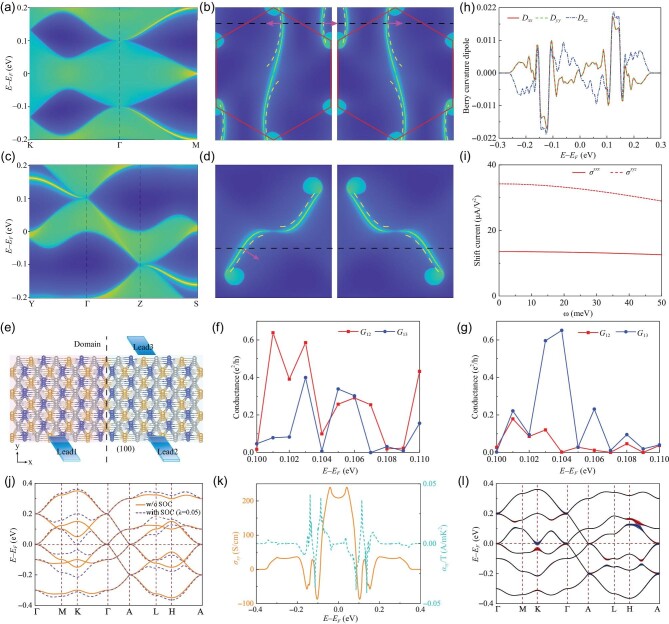
(a) and (c) The (001) and (100) surface spectral functions along specific paths. (b) and (d) Isoenergetic contours in the (001) and (100) surface BZs, simulated at 0.1 eV (double Weyl point at Γ). The arrows represent the direction of the current transport at the interface (domain wall) on the (001) and (100) planes. Solid curves are Fermi arcs at 0.1 eV, while dashed curves are those at a slightly higher energy. (e) Schematic diagram of non-local electrical transport for finite-size materials and a three-terminal device. (f) and (g) The conductance of devices with uniform chirality and opposite chiralities (corresponding to (e)) in the energy range of (100) surface states close to the double Weyl point, respectively. Here *G*_12(3)_ is the conductance between Lead1 and Lead2(3). (h) Berry curvature dipole as a function of the Fermi level. (i) Photo-energy-dependent shift current at 0.1 eV. (j) The band structures of a magnetic double Weyl semimetal without and with SOC based on Fig. 1(h), where Zeeman splitting and SOC strength are taken as *m* = 0.1 and λ = 0.05. (k) AHC and ANC as a function of the Fermi level and (l) the band structure weighted by Berry curvature, where blue and red represent negative and positive Berry curvatures, respectively, for a magnetic double Weyl semimetal with SOC.

Without losing generality, we analyze the topological properties of the double Weyl state in Fig. 1(h) as an example, as well as the corresponding Hall response and photocurrent. Beautiful Fermi arcs that start from points Γ or A and cross the Brillouin zone (BZ) boundary are observed in panels (a) and (c) of Fig. 2, which exhibit the topological surface states corresponding to Fig. 1(h) on the semi-infinite planes (001) and (100), respectively. The simulated open isoenergetic counters at 0.1 eV on the (001) and (100) planes, in which the time-reversed pairs of Fermi arcs are obtained, are given in panels (b) and (d) of Fig. 2. The presence of Fermi arcs is also verified by a loop along the specific path, which shows a projected Chern number of |*C*| = 1. In particular, a sizable topologically non-trivial energy window with a chiral helical state is observed, as shown in Fig. S1 within the online supplementary material. The reason for observing only one Fermi arc and one chiral surface state is because SOC is not considered here. In the presence of SOC, two chiral surface states with a topological charge of 2 will manifest, validating the double Weyl point, which will be further discussed below. The subtle surface states endow the 3D triangular lattice with unique transport properties. If a current is incident on the left surface on the (001) plane, part of the current will be reflected, while the remainder will flow through the domain wall and experience refraction. On the (100) plane, however, the absence of corresponding scattering channels prevents the current incident from the left surface from being reflected or refracted into its right enantiomorph. Therefore, the current can only be transmitted along the interface (domain wall) until it reaches another surface. Since this lattice is a chiral system, two materials with different chiralities can be naturally grown. By joining the proper terminal surfaces of two different handed materials to create the domain wall, realizing the unconventional transport stated above is extremely promising.

To confirm the non-local electrical transport between two open surfaces with opposite chiralities through the domain wall in a 3D triangular lattice, we design a three-terminal finite electronic device as shown in Fig. 2(e). The semi-infinite metals Lead1 and Lead2 are connected in the (100) plane and separated by a domain wall. Lead3 is located on the ($\bar{1}$00) plane and shares the same chirality as Lead2. Here, Lead1 is configured as the current incoming terminal, while Lead2 and Lead3 are set as the outgoing terminals. To directly reveal the impact of surfaces with opposite chiralities on the electronic transport, we calculate the conductance of two devices in the energy range of (100) surface states near the double Weyl point (see the calculation method in the online supplementary material), in which one device is uniformly chiral, while the other consists of two regions with opposite chiralities (see Fig. 2(e)), each of which is a 12 × 10 × 10 supercell. When the 3D triangular device has uniform chirality, the conductances *G*_12_ and *G*_13_ fluctuate with the change of energy, but *G*_12_ is larger than *G*_13_ below 0.104 eV, as seen in Fig. 2(f), which shows that the incoming current from Lead1 prefers to partition into Lead2. This is due to Lead2 being positioned closer to Lead1 than Lead3. However, when the device has opposite chiralities (see Fig. 2(e)), *G*_13_ will surpass the value of *G*_12_. When the Fermi energy is located at 0.102–0.104 eV, *G*_13_ is much greater than *G*_12_, as depicted in Fig. 2(g). These findings undoubtedly illustrate the non-local electrical transport associated with topological surface states in a 3D triangular lattice.

### Non-linear Hall effect

The non-magnetic 3D triangular lattice with TR symmetry has a total Berry curvature integral of zero, which causes Hall conductance to vanish. However, due to spontaneous inversion symmetry breaking, which permits the dipole moment of Berry curvature to be non-zero, a NLH conductance can be produced. We consider the maximum point group 32 corresponding to SGs No. 154 and No. 152 that enables the 3D triangular lattice. Under the symmetry constraint, only the diagonal terms of the BCD deviate from zero. The calculated BCD for the double Weyl point is shown in Fig. 2(e), where *D_xx_* = *D_yy_* ≠ *D_zz_* is obtained. It is also noted that the BCD exhibits a negative maximum near the double Weyl Γ point (0.1 eV), while the BCD reaches a positive maximum at the energy slightly higher than 0.1 eV, reflecting the characteristics of complex band edges in metals.

Next, we discuss a possible experimental observable corresponding to BCD. In general, the second-harmonic part of the NLH current related to BCD can be represented by ${j_a^{2\omega }=\chi _{abc}E_b^{\omega }E_c^{\omega }}$, where χ_*abc*_ is the non-linear tensor and *E* is an external electric field. In a TR invariant system [28], χ_*abc*_ = ε^*acd*^*D_bd_e*^3^τ/2ℏ^2^(1 + *i*ωτ), where τ is the relaxation time and ε denotes the Levi-Civita symbol. BCD with the symmetry constraint has only three non-zero matrix elements, *D_xx_* = *D_yy_* and *D_zz_*, so that possible non-linear tensor elements are χ_*yxz*_ = χ_*zyx*_ = −χ_*xyz*_ = −χ_*zxy*_ = *d*_1_ and χ_*yzx*_ = −χ_*xzy*_ = *d*_2_. Thus, for an electric field in the *x*–*z* plane, we have $j_y^{(2)}\sim D_{xx}E_xE_z+D_{zz}E_xE_z$, i.e. the non-linear Hall current can be measured in the *y* direction. Based on symmetry analysis, non-linear susceptibility has two independent non-zero elements *d*_1_ and *d*_2_. Here, coordinates *x* and *z* are taken as the *a* and *c* axes of the crystal. For an electric field *E* = (*E_x_*, 0, *E_z_*), the non-linear current density **j**^(2)^ is given as


\begin{eqnarray*}
\mathbf {j}^{(2)}= \left({\begin{array}{c}0 \\
2(d_1+d_2)E_xE_z \\
0 \end{array}}\right).
\end{eqnarray*}

According to Ohm’s law, we have the second-order non-linear electric field


\begin{eqnarray*}
\mathbf {E}^{(2)}=\left({\begin{array}{c}0 \\
2(d_1+d_2)\rho _bE_xE_z\\
0 \end{array}}\right).
\end{eqnarray*}

For the applied current in the *x*–*z* plane,


\begin{eqnarray*}
\mathbf {j} = j \!\!\left({\begin{array} {c} {\mathrm{sin}}\theta \\
0 \\
{\rm cos}\theta
\end{array}}\right),
\end{eqnarray*}

where *j* is the current amplitude and θ is the angle measured from the *c* axis, we have the first-order electric field


\begin{eqnarray*}
\mathbf {E}=j \!\!\left({\begin{array} {c} \rho _a {\rm sin}\theta \\
0 \\
\rho _c {\rm cos}\theta
\end{array}}\right),
\end{eqnarray*}

and its longitudinal component is *E*_‖_ = *j*(ρ_*a*_sin^2^θ + ρ_*c*_cos^2^θ). Moreover, the transverse component of the second-order electric field can be written as $\smash{E_{\bot }^{(2)}}=j^2(d_1+d_2)\rho _c^3\gamma _1 \gamma _2 {\rm sin}2\theta$, where γ is the resistance anisotropy as γ_1_ = ρ_*a*_/ρ_*c*_ and γ_2_ = ρ_*b*_/ρ_*c*_. Thus, an angle-resolved non-linear response through the second-order non-linear susceptibilities can be obtained as


(3) \begin{eqnarray*}
\frac{V_\bot ^{2\omega }}{V_{\Vert }^2}=\rho _c {\rm sin}2\theta \frac{(d_1+d_2)\gamma _1\gamma _2}{(\gamma _1{\rm sin}^2\theta +{\rm cos}^2\theta )^2}.
\end{eqnarray*}

According to the global factor sin2θ, it can be inferred that the NLH response is maximal with the applied driving current along the diagonal direction of the *x*–*z* plane. Regarding the point group 3 corresponding to SGs No. 145 and No. 144, in addition to the diagonal terms of the BCD, the off-diagonal term *D_xy_* is also non-zero, resulting in quite different NLHEs (see the online supplementary material).

### Photovoltaic effect

The 3D triangular lattice only exists in a chiral system with broken inversion and mirror symmetries, which is capable of supporting a quantized CPGE and shift current. Since quantized CPGE is only related to the topological charge of Weyl points and is independent of individual materials, for a double Weyl semimetal, if we fix the chemical potential at a double Weyl point, a quantized CPGE β(ω) = *iC*π*e*^3^/*h*^2^ with |*C*| = 2 (charge of the double Weyl node) can be obtained. What we are concerned with here is the shift current, which is connected to the specifics of the energy bands. We set the chemical potential at the double Weyl Γ point (0.1 eV) to concentrate on the optical dc conductivity of the 3D triangular lattice. The optical dc-conductivity shift current from the contribution of the double Weyl node in the 3D triangular lattice is plotted in Fig. 2(f), in which $J_{SC}^i = 2\sigma ^{ijk}E^jE^k$ is the shift current along the *i* direction. Only two independent tensor elements σ^*xyz*^ and σ^*xxx*^ are non-zero, and the other non-zero tensor elements can be obtained using symmetry, such as σ^*yxz*^ = −σ^*xyz*^. From Fig. 2(f), we find that σ^*xyz*^ can reach as high as 30 *μ*A/V^2^ and is more than twice σ^*xxx*^, and both of them show parabolic dependency on photoenergy.

### Anomalous Hall effect and anomalous Nernst effect

To study AHE and ANE, we consider a ferromagnetic system—a magnetic double Weyl semimetal based on Fig. 1(h), where a Zeeman term *m* = 0.1 is added to Equation (1). The band structures without and with SOC are given in Fig. 2(j). Here, we take the SOC strength as λ = 0.05. In real materials, SOC arises from the out-of-plane electric field due to the atoms located inside the triangles of the 3D triangular lattice. Thus, it usually gives the dominated SOC form. However, the electric field that generates SOC does not have to be out of plane, that is, the SOC caused by the in-plane electric field can also generate AHE and ANE (see Fig. S2 within the online supplementary material). SOC opens tiny band gaps at several crossing points, resulting in finite AHC, as shown in Fig. 2(k). ANE is the creation of transverse electric voltage that is perpendicular to both magnetization and an applied temperature gradient in a magnet. ANE is proportional to magnetization and is driven by Berry curvature or the non-trivial geometric structure. ANC α_*xy*_ is given by the Mott relation [32]


\begin{eqnarray*}
\frac{\alpha _{xy}}{T}=\frac{\pi ^2k_B^2}{3e}\frac{d}{d\epsilon }[\sigma _{xy}(\epsilon )]_{\mu },
\end{eqnarray*}

where *k_B_* is the Boltzmann constant and μ is the chemical potential. As seen in Fig. 2(h), AHC changes dramatically near the small band gaps opened by SOC, resulting in several peaks of ANC. To unveil the nature of large AHC, we depict the band structure with SOC weighted by the magnitude of Berry curvature Ω_*xy*_(**k**) in Fig. 2(l). It is obvious that Berry curvature is predominantly distributed at double Weyl Γ and A points and the band edges of SOC-induced band gaps, especially at the band edges of a small band gap. The large Berry curvature distributed at the band edges of SOC-induced band gaps of Weyl points, which results in AHE, is a special property of the magnetic Weyl semimetal [7]. Here, the special property for a 3D triangular lattice is that it can naturally generate various Weyl semimetal states. As a result, in a magnetic 3D triangular lattice, the finite SOC can induce a small band gap at Weyl points, which will produce a large Berry curvature, giving rise to a large AHC. Considering the inherent topological properties of the 3D triangular lattice and the intrinsic SOC effect in real materials, the magnetic 3D triangular lattice is very suitable for studying AHE and ANE.

## REALISTIC MATERIALS

As we previously mentioned, there are four space groups satisfying the 3D triangular lattice with different hopping parameters. Checking the Inorganic Crystal Structure Database (see https://icsd.fiz-karlsruhe.de/index.xhtml), we find a large number of experimentally synthesized materials that hold a 3D triangular crystalline lattice, which display different topological phases as we discussed in Fig. 1. Here, we choose two of them (one non-magnet and one ferromagnet) as examples for in-depth discussion, and we also list some other systems in the online supplementary material that merit further research.

### Non-magnetic triangular lattice for SHE, NLHE and the shift current

**Figure 3.**
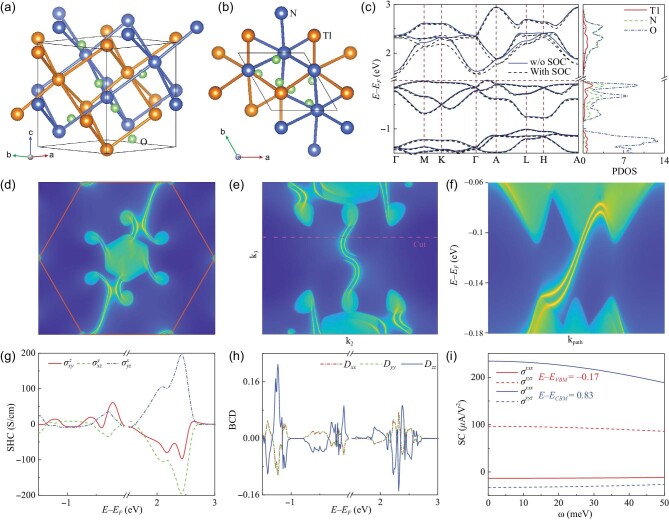
(a) Side and (b) top views of the crystal structure for TlNO_2_, where Tl and N atoms constitute a 3D triangular lattice, respectively. (c) Electronic band structures of TlNO_2_ without/with SOC and projected density of states (PDOS) with SOC. Calculated Fermi arc surface sates of the (d) (001) and (e) (100) surfaces of TlNO_2_. ‘Cut’ is a line cut across the surface BZ in the *k_z_* direction, which corresponds to a 2D *k_x_*–*k_y_* slice. (f) The *E* − *k* dispersion along the ‘cut’, indicated by the dashed line in (e). The yellow lines denote surface states and the shaded areas are the projections of the bulk bands. (g) SHC and (h) BCD as a function of the Fermi level for TlNO_2_. (i) Shift current as a function of photoenergy at energies of 0.17 meV below VBM and 0.83 meV above CBM.

The crystal structure of TlNO_2_ with SG No. 152 is given in Fig. 3(a) and (b), where Tl and N atoms form a 3D triangular lattice, respectively. The triangles in Fig. 3(b) differ slightly in length from an equilateral triangle, and on the other hand, the distribution of O atoms within the triangles is not uniform, bringing about different NN hopping parameters that are consistent with our model. Therefore, we obtain the kagome bands and bands of phase A in the *k_z_* = 0 and π planes of both conduction bands (CBs) and valence bands (VBs), respectively, as seen in Fig. 3(c). It is noted that the electronic states of CB and VB close to the Fermi level are dominated by N and O atoms (i.e. *p* orbital). The spin-polarized calculations show that this material has a non-magnetic ground state, and SOC leads to more significant splitting in CB than VB. The Weyl points at K and H and double Weyl points at Γ and A are the most noticeable topological features. A crucial indicator of the Weyl nodes is the existence of Fermi arcs. We calculate the (001) and (100) surface states in panels (d) and (e) of Fig. 3, respectively, which unmistakably demonstrate the existence of two Fermi arcs. This is in line with its two chiral charges. These two Fermi arcs are clearly separated from one another in *k* space, indicating that SOC cannot be ignored even for *p*-orbital electrons [35]. The energy dispersion of the surface states along the ‘cut’ (red dashed line in Fig. 3(e)) is depicted in Fig. 3(f), where two copropagating surface states emerge in the expansive energy gap window associated with Chern number 2 of the 2D *k* slice.

The non-negligible SOC can mix different spin components of wave functions and induce an appreciable SHC. The three independent components of the SHC tensor, $\sigma _{xy}^z$, $\sigma _{xz}^y$ and $\sigma _{yz}^x$, are calculated as shown in Fig. 3(g). We find that after being doped with some electrons and holes, the system can generate a considerable SHC. In particular, after doping some electrons, SHC can reach about 200 S/cm, which is comparable to or even larger than that of many heavy element systems with a large SOC [36–38]. Because of the inversion asymmetric structure, the distribution of Berry curvature is not uniform, producing a non-zero BCD, as shown in Fig. 3(h). The anisotropic Berry curvature and velocity are discovered to produce a substantial BCD near the Weyl points, which typically exhibit a large Berry curvature. In addition, the finite Berry curvature can also reveal a non-zero shift current, as presented in Fig. 3(i). It is noted that if we fix μ at the Weyl points, i.e. 0.17 eV below the VB maximum (VBM) and 0.83 eV above the CB minimum (CBM), we can get a considerable shift current, where σ^*xxx*^ can attain more than 200 ${\rm \mu A/V^2}$ at *E* − *E*_CBM_ = 0.83 eV.

### Ferromagnetic triangular lattice for AHE and ANE

**Figure 4.**
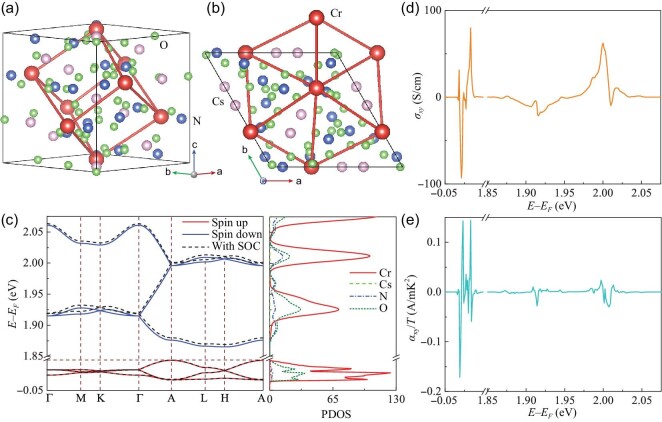
(a) Side and (b) top views of the crystal structure for CrCs_2_(NO_3_)_5_ with a space group of 154. (c) The spin-polarized band structure of CrCs_2_(NO_3_)_5_ without and with SOC and corresponding PDOS with SOC. (d) AHC and (e) its thermoelectric counterpart ANC as a function of the Fermi level.

Now we introduce a 3D ferromagnetic triangular lattice. The crystal structure of CrCs_4_(NO_3_)_5_ with SG No. 154 is presented in Fig. 4(a) and (b), with Cr atoms forming a 3D triangular lattice. The inhomogeneous distribution of Cs, N and O atoms in the 3D periodic lattice gives rise to different NN hoppings. This system displays ferromagnetic ground states, according to spin-polarized calculations. The band structure in Fig. 4(c) indicates that the spin-down and spin-up electrons of Cr atoms are preponderant at CB and VB, respectively. Because of the quenching of the *d*-orbital angular momentum of Cr atoms, the SOC is fairly weak in CrCs_4_(NO_3_)_5_. Even so, we still achieve the maximum AHC close to 100 S/cm within 30 meV below the Fermi level, as seen in Fig. 4(d). The calculated ANC is given in Fig. 4(e). Within 30 meV below the Fermi level, we find several pronounced peak-valley structures. This oscillating behavior originates from the intricate narrow band structure and occurs at a spot where AHC has a sudden change of sign and magnitude. The highest α_*xy*_/*T* can reach 0.17 A/mK^2^, which is more than an order of magnitude higher than many documented systems [39–41]. This indicates the feasibility of employing the magnetic material CrCs_4_(NO_3_)_5_ and its band topology for spin caloritronics applications.

Here, we would like to emphasize that the two materials we discussed above were only used to demonstrate the effectiveness of our proposed lattice model, and some of their properties may not be optimal. We also list a few systems with various topological band structures of a 3D triangular lattice in Fig. S3 within the online supplementary material, where systems with better performance may be found, which is worthy of further in-depth exploration. In addition, the materials we listed have been synthesized experimentally, and we look forward to exploring these systems in future experiments.

Here we would like to remark that our proposed 3D triangular lattice is versatile and can realize different topological phenomena in one simple model, demonstrating its uniqueness owing to a few essential features. Firstly, such a 3D lattice is a chiral lattice, which is different from the widely studied 2D lattices with the centrosymmetric structure. The chiral lattice can provide the basis for studying the Weyl fermion physics, and is also essential for the study of many unique topological properties, such as NLHE, topological photocurrent, etc. Besides, in contrast to 2D lattice models that usually hold high-order NN hoppings, the relatively large atomic spacing in our 3D triangular lattice is calibrated to make next-NN hopping negligible. As a result, compared with other lattices this 3D triangular lattice is not only a simple lattice model, but can also be applicable to many realistic material systems. More importantly, our 3D triangular lattice has a special geometric structure with a spiral triangular lattice along the *z* axis, which is the origin of those topological nodal points, leading to the versatility in describing different topological phenomena.

## CONCLUSION

In this work, we present a general model in a 3D triangular lattice with alternating hopping parameters, which can generate a variety of intriguing topological phases, including kagome bands, triply degenerate fermions, double Weyl fermions, and others. This effective lattice model is demonstrated to display many fascinating Berry-curvature-related phenomena, such as AHE, SHE, ANE, unconventional transport, NLHE, the shift current and so on. In addition, this lattice model has been shown to be applicable to many materials that have been synthesized experimentally. We identify a few materials with various topological band structures and discuss two of them in detail. Our work not only unifies numerous physical phenomena related to Berry curvature in one simple model, but also offers a wealth of real material examples for further theoretical investigation and device applications. Apart from those topological effects we have discussed in this article, this model can also be applied to the study of chiral anomaly, other nonlinear effects, etc., which will stimulate more theoretical and experimental studies in the future.

## Supplementary Material

nwad114_Supplemental_File
