# Supplementary material for: A versatile model with three-dimensional triangular lattice for unconventional transport and various topological effects
Source: Natl Sci Rev. 2023 Apr 25;11(1):nwad114. doi: 10.1093/nsr/nwad114 (PMC10727845; doi:10.1093/nsr/nwad114)
Supplement: nwad114_Supplemental_File [file nwad114_supplemental_file.pdf]

Supplementary Data for "A versatile model with three-dimensional triangular lattice for unconventional transport and various topological effects"

Jing-Yang You,[1] Gang Su,[2] and Yuan Ping Feng[1,3]

[1]*Department of Physics, National University of Singapore, 2 Science Drive 3, Singapore 117551*

[2]*Kavli Institute for Theoretical Sciences, and CAS Center for Excellence in Topological Quantum Computation, University of Chinese Academy of Sciences, Beijing 100190, China*

[3]*Centre for Advanced 2D Materials, National University of Singapore, 6 Science Drive 2, Singapore 117546*

Calculation methods

Our first-principles calculations for real materials were based on density functional theory (DFT) as implemented in the Vienna Ab initio Simulation Package (VASP) [1], using the projector augmented-wave method [2]. The generalized gradient approximation with the Perdew-Burke-Ernzerhof [3] realization was adopted for the exchange-correlation functional. The plane-wave cutoff energy was set to 550 eV. A Monkhorst-Pack k-point mesh [4] with a size of 13×13×13 was used for the BZ sampling. The crystal structure was optimized until the forces on the ions were less than 0.001 eV/Å. The surface spectrum was calculated by using the Wannier functions and the iterative Green's function method [5–7]. The non-equilibrium Keldysh Green's function and Landauer-Bttiker formula [8] as implemented in the numerical package KWANT [9] were employed to calculate the electronic conductance G, i.e. $G_{12} = (2e^2/h)\mathrm{Tr}(\Gamma_2 G^r \Gamma_1 G^a)$, where G_{12} represents the conductance from Lead1 to Lead2, $G^{r/a}$ are the retarded and advanced Green's functions of the central lattice region, and $\Gamma_{1/2}$ are the line-width functions that couple Lead1 and Lead2 terminals to the central region, respectively.

The anomalous Hall conductivity was calculated via:

$$\sigma_{\alpha\beta} = -\frac{e^2}{\hbar}\epsilon_{\alpha\beta\gamma}\sum_n \int \frac{d\mathbf{k}}{(2\pi)^3} f_{n\mathbf{k}}\Omega_n^{\gamma}(\mathbf{k}), \tag{S1}$$

where $f_{n\mathbf{k}}$ is the Fermi distribution function and $\Omega_n(\mathbf{k}) = \nabla_k \times i\langle n,\mathbf{k}|\nabla_k|n,\mathbf{k}\rangle$ is the Berry curvature [10]. According to the Kubo formula, the intrinsic spin Hall conductivity (SHC) can be written as [11–13]

$$\sigma_{xy}^{\mathrm{spinz}}(\omega) = \hbar \int \frac{d^3k}{(2\pi)^3}\sum_n f_{n\mathbf{k}} \sum_{m\neq n} \frac{2\mathrm{Im}[\langle n\mathbf{k}|\hat{j}_x^{\mathrm{spinz}}|m\mathbf{k}\rangle\langle m\mathbf{k}| - e\hat{v}_y|n\mathbf{k}\rangle]}{(\epsilon_{n\mathbf{k}} - \epsilon_{m\mathbf{k}})^2 - (\hbar\omega + i\eta)^2}, \tag{S2}$$

where n and m are band indexes, ϵ_n and ϵ_m are the eigenvalues, $\hat{j}_x^{\mathrm{spinz}} = \frac{1}{2}\{\hat{s}_z, \hat{v}_x\}$ is the spin current operator and $\hat{s}_z = \frac{\hbar}{2}\hat{\sigma}_z$ is the spin operator, $\hat{v}_y = \frac{1}{\hbar}\nabla_y H(\mathbf{k})$ is the velocity operator, and the frequency ω and η are set to zero in the dc clean limit. It is noted that if we replace $\hat{j}_x^{\mathrm{spinz}}$ in Eq. S2 by $-e\hat{v}_x$, Eq. S2 is exactly Eq. S1. The Berry curvature dipole that describes nonlinear Hall effect is defined as [14]

$$D_{ab} = \int_k f_0 \partial_a \Omega_b. \tag{S3}$$

The shift current was calculated by Wannier interpolation method [15] based on the formula [16]:

$$\sigma^{abc}(0;\omega,-\omega) = -\frac{i\pi e^3}{4\hbar^2}\int_k \sum_{n,m} f_{nm}(I^{abc}_{mn} + I^{acb}_{nm})[\delta(\omega_{mn}-\omega)+\delta(\omega_{nm}-\omega)], \quad \text{(S4)}$$

where $I^{abc}_{mn} = r^b_{mn} r^{c;a}_{nm}$, $r^a_{\mathbf{k}nm} = (1-\delta_{nm})A^a_{\mathbf{k}nm}$, $r^{a;b}_{\mathbf{k}nm} = \partial_b r^a_{\mathbf{k}nm} - i(A^b_{\mathbf{k}nn} - A^b_{\mathbf{k}mm}) r^a_{\mathbf{k}nm}$, $A^a_{\mathbf{k}nm} = i\langle u_{\mathbf{k}n}|\partial_a u_{\mathbf{k}m}\rangle$ is the Berry connection matrix, $f_{nm} = f_n - f_m$ and $\hbar\omega_{nm} = E_m - E_n$ are differences between occupation factors and band energies, respectively.

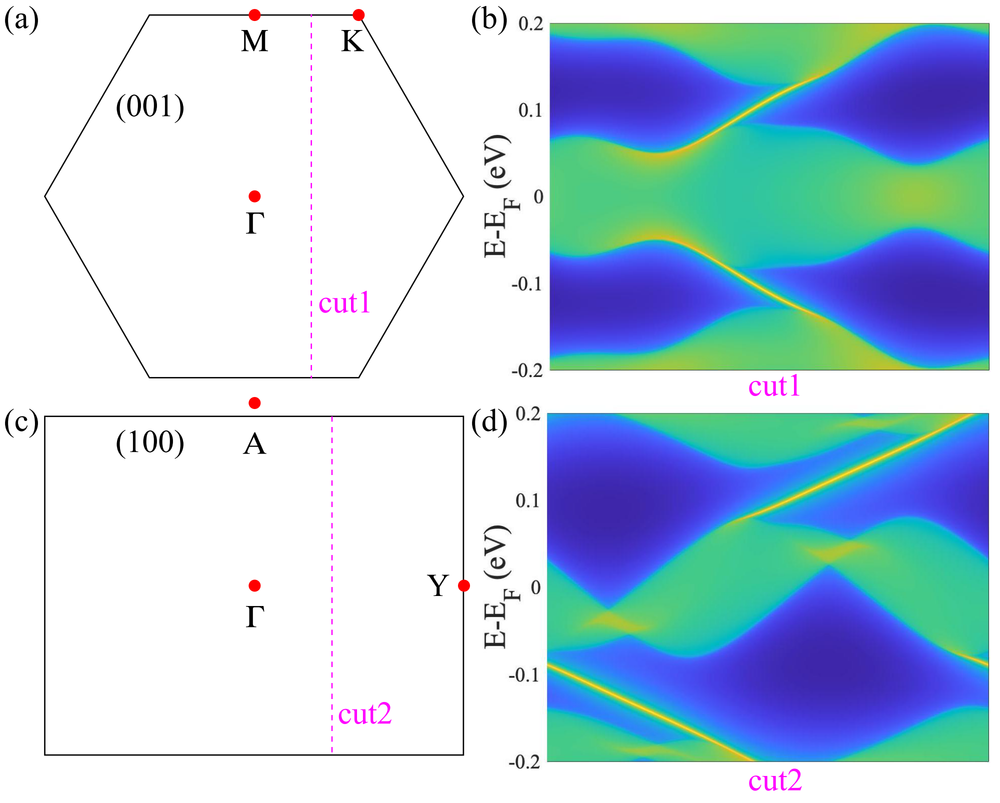

FIG. S1. (a) and (c) The surface BZs for (001) and (100) surfaces, respectively. (b) and (d) The surface spectral functions along the k paths cut1 and cut2 highlighted in (a) and (c) for (001) and (100) surfaces, respectively.

Symmetry analysis

In this section, we will give a symmetry analysis of why the Weyl points locate at the high-symmetry points in our model and take K and Γ points as examples. The $k\cdot p$ model $\mathcal{H}$

is subjected to the D_3 little group at K, with two generators, C_{3z} and C_{2x}. The symmetry constraints are given by

$$C_{3z}^{-1}\mathcal{H}(\mathbf{q})C_{3z}=\mathcal{H}(C_{3z}\mathbf{q})=\mathcal{H}(-q_x/2-\sqrt{3}q_y/2,\sqrt{3}q_x/2-q_y/2,q_z), \tag{S5}$$

$$C_{2x}^{-1}\mathcal{H}(\mathbf{q})C_{2x}=\mathcal{H}(C_{2x}\mathbf{q})=\mathcal{H}(q_x,-q_y,-q_z), \tag{S6}$$

where $\mathbf{q}$ is measured from K. In the basis of the 2D irreducible representation E for D_3, we find that to linear order in $\mathbf{q}$, the effective model takes the form of the 2D Weyl model,

$$\mathcal{H}(\mathbf{q})=a_0+\nu_1(q_x\sigma_z-q_y\sigma_x)+\nu_2 q_z\sigma_y, \tag{S7}$$

where ν_1 and ν_2 are the Fermi velocities in the xy plane and z direction, respectively, and $\sigma_{x/y/z}$ are the Pauli matrices acting in the space of the two basis states. Thus, the low-energy electrons indeed resemble 2D Weyl fermions.

As for Γ point, it not only have the D_3 little group, but also is a time-reversal invariant momentum. The time-reversal symmetry constraint is given by

$$\mathcal{T}^{-1}\mathcal{H}\mathcal{T}=\mathcal{H}(\mathcal{T}\mathbf{q})=\mathcal{H}(-\mathbf{q}), \tag{S8}$$

where $\mathbf{q}$ is measured from Γ. In the basis of the 2D irreducible representation E for D_3, we find that to leading order in $\mathbf{q}$, the effective model takes the form

$$\mathcal{H}(\mathbf{q})=f_0(\mathbf{q})\sigma_0+\sum_{i=x,y,z}f_i(\mathbf{q})\sigma_i, \tag{S9}$$

where $f_0(\mathbf{q})=a_0+a_1(q_x^2+q_y^2)+a_2q_z^2$, $f_x(\mathbf{q})=-2b_1q_xq_y+b_2q_xq_z$, $f_y(\mathbf{q})=c_0$ and $f_z=-b_1(q_x^2-q_y^2)+b_2q_yq_z$. The reduced Hamiltonian is in the Weyl form, linear in q_x, q_y, and q_z at K but has the leading second-order terms of q_x, q_y, and q_z at Γ. Similar results can be obtained at H and A points. These results indicate that two Weyl points with topological charges ±1 can be found at K and H and double Weyl points with topological charges ±2 can be found at Γ and A. The topological charge 2 of double Weyl points are protected by the combination of the screw symmetry and the time-reversal symmetry.

Non-linear Hall effect for the space groups of No. 144 and 145

In general, the second-harmonic part of the non-linear Hall current related to BCD can be represented by $j_a^{2\omega}=\chi_{abc}E_b^{\omega}E_c^{\omega}$, where χ_{abc} is the non-linear tensor and the E is an external

electric field. In the TR invariant system, $\chi_{abc} = \epsilon^{acd} D_{bd} \frac{e^3\tau}{2\hbar^2(1+i\omega\tau)}$, where τ is the relaxation time and ϵ is the Levi-Civita symbol. The space groups of No. 144 and 145 correspond the point group of 3. BCD with symmetry constraint has the non-zero matrix elements D_{xx}, D_{yy}, D_{zz} and $D_{yx} = -D_{xy}$, so that possible non-linear tensor elements are $\chi_{yxz} = -\chi_{zxy} = d_1$, $\chi_{zyx} = -\chi_{xyz} = d_2$, $\chi_{yzx} = -\chi_{xzy} = d_3$ and $\chi_{zxx} = -\chi_{xxz} = \chi_{zyy} = -\chi_{yyz} = d_4$. Thus, for an electric field in the x direction, we have $j_z^{(2)} \sim D_{xy} E_x E_x$, i.e. the non-linear Hall current can be measured in the z direction. Based on the symmetry analysis, the non-linear susceptibility has four independent non-zero elements d_i $(i = 1, 2, 3, 4)$. Here, the coordinates x and z are taken as the a and c axes of the crystal. For an electric field $E = (E_x, 0, E_z)$, the nonlinear current density $\mathbf{j}^{(2)}$ is given as $\mathbf{j}^{(2)} = \begin{pmatrix} -2d_4 E_x E_z \\ 2(d_1 + d_3) E_x E_z \\ d_4 E_x^2 \end{pmatrix}$. According to the Ohm's law, we have the second-order non-linear electric field $\mathbf{E}^{(2)} = \begin{pmatrix} -2d_4 \rho_a E_x E_z \\ 2(d_1 + d_3) \rho_b E_x E_z \\ d_4 \rho_c E_x^2 \end{pmatrix}$. For the applied current in xz plane, $\mathbf{j} = j \begin{pmatrix} \sin\theta \\ 0 \\ \cos\theta \end{pmatrix}$, where j is the current amplitude and θ is the angle measured from c axis, we have the first order electric field $\mathbf{E} = j \begin{pmatrix} \rho_a \sin\theta \\ 0 \\ \rho_c \cos\theta \end{pmatrix}$, and its longitudinal component is $E_\parallel = j(\rho_a \sin^2\theta + \rho_c \cos^2\theta)$. If the non-linear Hall effect is measured in the y direction, the angle resolved non-linear response takes the similar form as Eq.(4) in the main text. If the non-linear Hall effect is measured in xy plane, the transverse component of the second-order electric field can be written as $E_\perp^{(2)} = j^2 d_4 \rho_c^3 \sin\theta (1 + \gamma_1^2 \cos^2\theta)$, where γ_1 is the resistance anisotropy as $\gamma_1 = \rho_a/\rho_c$. Thus, angle resolved non-linear response through the second-order non-linear susceptibilities can be obtained as:

$$\frac{V_\perp^{2\omega}}{V_\parallel^2} = d_4 \rho_c \sin\theta \frac{(1 + \gamma_1^2 \cos^2\theta)}{(\gamma_1 \sin^2\theta + \cos^2\theta)^2}. \tag{S10}$$

According to the global factor $\sin\theta$, it can be inferred that the non-linear Hall response is maximal when the driving current is applied along the x direction.

Anomalous Hall and Nernst effects with the SOC from an in-plane electric field

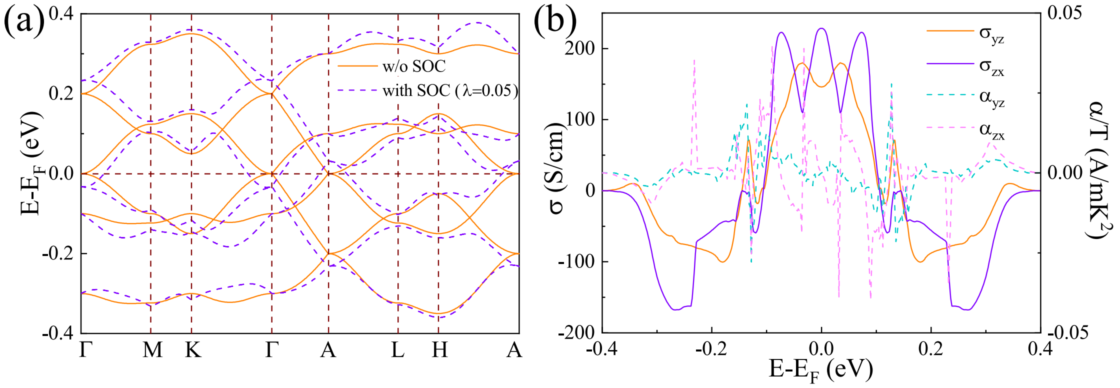

FIG. S2. The band structures of magnetic double Weyl semimetal without and with SOC from an in-plane electric field based on Fig. 1(h), where Zeeman splitting and SOC strength are taken as $m = 0.1$ and $\lambda = 0.05$. (b) The AHC and ANC as a function of Fermi level.

In Fig. S2(a), we plot the band structures of magnetic double Weyl semimetal without SOC and with an in-plane electric field induced SOC. The SOC also opens tiny band gaps at several crossing points, resulting in finite AHC as shown in Fig. S2(b). AHC changes dramatically near the small band gaps opened by SOC, resulting in several peaks of ANC. Compared to the case with the SOC caused by an out-of-plane electric field in Fig. 2(h), where the AHC and ANC are measured in the xy plane, in the presence of an in-plane electric field induced SOC, the AHC and ANC are measured in the xz and yz planes.

Material candidates

Besides the two 3D triangular materials discussed in the main text, some other 3D triangular materials are also shown in Fig. S3.

[1] G. Kresse and J. Furthmüller, Phys. Rev. B **54**, 11169 (1996).

[2] P. E. Blöchl, Phys. Rev. B **50**, 17953 (1994).

[3] J. P. Perdew, K. Burke, and M. Ernzerhof, Phys. Rev. Lett. **77**, 3865 (1996).

[4] H. J. Monkhorst and J. D. Pack, Phys. Rev. B **13**, 5188 (1976).

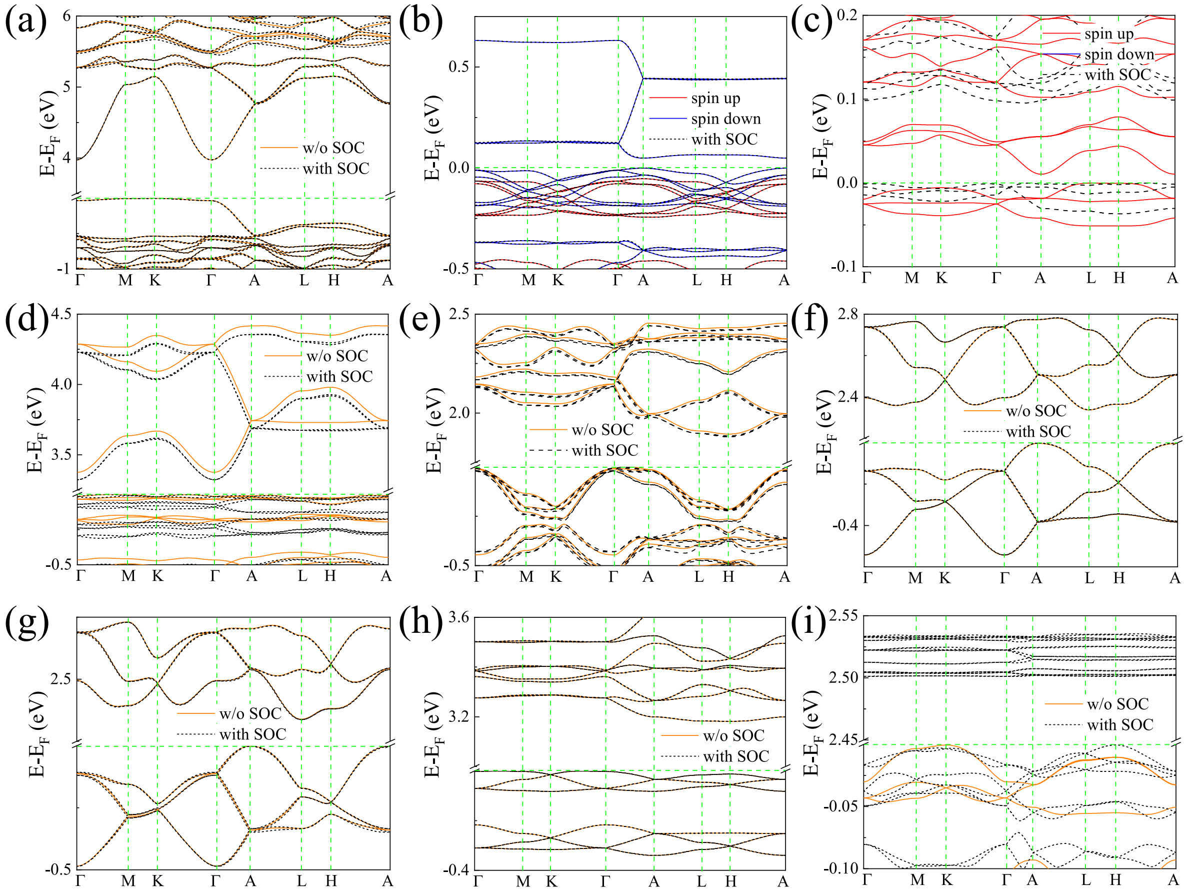

FIG. S3. The electronic band structures of several experimentally synthesized 3D chiral triangular materials, including (a) $PrGeBO_5$ (86744), (b) ferromagnetic (FM) $Cr_2K_2AsO_{10}$ (30533) and (c) FM $CeSiBO_5$ (28026) in SG 144, (d) $HgCNCl_3$ (109499) and (e) Cr_2HgO_7 (416169) in SG 145, (f) $RbNO_2$ (35102), (g) $CsNO_2$ (38068), (h) $Rb_2Sc(NO_3)_5$ (400315) and (i) $La(AlBr_4)_3$ (72281) in SG 152.

[5] N. Marzari and D. Vanderbilt, Phys. Rev. B **56**, 12847 (1997).

[6] I. Souza, N. Marzari, and D. Vanderbilt, Phys. Rev. B **65**, 035109 (2001).

[7] M. P. L. Sancho, J. M. L. Sancho, J. M. L. Sancho, and J. Rubio, J. Phys. F: Met. Phys. **15**, 851 (1985).

[8] S. Datta, *Electronic Transport in Mesoscopic Systems* (Cambridge University Press, 1995).

[9] C. W. Groth, M. Wimmer, A. R. Akhmerov, and X. Waintal, New J. Phys. **16**, 063065 (2014).

[10] N. Nagaosa, J. Sinova, S. Onoda, A. H. MacDonald, and N. P. Ong, Rev. Modern Phys. **82**, 1539 (2010).

[11] Y. Yao and Z. Fang, Phys. Rev. Lett. **95**, 156601 (2005).

[12] J. Qiao, J. Zhou, Z. Yuan, and W. Zhao, Phys. Rev. B **98**, 214402 (2018).

[13] J. H. Ryoo, C.-H. Park, and I. Souza, Phys. Rev. B **99**, 235113 (2019).

[14] I. Sodemann and L. Fu, Phys. Rev. Lett. **115**, 216806 (2015).

[15] J. E. Sipe and A. I. Shkrebtii, Phys. Rev. B **61**, 5337 (2000).

[16] J. Ibañez-Azpiroz, S. S. Tsirkin, and I. Souza, Phys. Rev. B **97**, 245143 (2018).
